# Reply to Yavuz, S. Comment on “Martire et al. Prognostic Role of High-Sensitivity C-Reactive Protein/Albumin Ratio in Heart Failure Patients. *Biomedicines* 2026, *14*, 748”

**DOI:** 10.3390/biomedicines14061282

**Published:** 2026-06-04

**Authors:** Domenico Martire, Giuseppe Armentaro, Giandomenico Severini, Carlo Alberto Pastura, Maria Rosangela Scarcelli, Velia Cassano, Martina Crasà, Ilaria Gareri, Gianluca Cortese, Valentino Condoleo, Raffaele Maio, Giorgio Sesti, Francesco Andreozzi, Angela Sciacqua

**Affiliations:** 1 Department of Medical and Surgical Sciences, University “Magna Graecia” of Catanzaro, 88100 Catanzaro, Italy; 2 Geriatrics Division, “Renato Dulbecco” University Hospital of Catanzaro, 88100 Catanzaro, Italy; 3 Department of Clinical and Molecular Medicine, University of Rome-Sapienza, 00189 Rome, Italy; giorgio.sesti@uniroma1.it; 4 Research Center for the Prevention and Treatment of Metabolic Diseases (RC-METDIS), University “Magna Graecia” of Catanzaro, 88100 Catanzaro, Italy

We would like to thank the author for the careful evaluation of our manuscript and for the thoughtful comments [1]. We welcome the opportunity to clarify several methodological aspects and to further strengthen the interpretation of our findings.

Single Baseline Measurement of hs-CRP and Serum Albumin: We acknowledge that hs-CRP and serum albumin are dynamic biomarkers that may be influenced by transient clinical conditions. However, it is important to emphasize that the use of baseline measurements for prognostic stratification is a widely accepted and consolidated approach in cardiovascular research, including studies involving established biomarkers such as natriuretic peptides. In this context, baseline hs-CRP and albumin values have been consistently shown to retain strong prognostic significance despite their biological variability. Specifically, hs-CRP is a validated marker of systemic inflammation and cardiovascular risk, mechanistically linked to endothelial dysfunction and atherothrombosis [2,3,4], while serum albumin reflects a composite of nutritional, inflammatory, and metabolic status and is independently associated with adverse outcomes in heart failure [5,6,7]. However, our study was designed as a retrospective cohort analysis, and repeated measurements were not consistently available. Future prospective studies are indeed warranted to evaluate the prognostic value of temporal changes in the hs-CRP/albumin ratio.

Use of Different Cut-Off Values (1.19 vs. 1.454): We appreciate the observation regarding the use of two thresholds. However, we would like to clarify that this approach was intentional and methodologically justified. The median value (1.19) was used for group stratification to ensure balanced sample sizes and statistical robustness in comparative analyses. In contrast, the ROC-derived cut-off (1.454) represents the optimal threshold for discriminative performance based on the Youden index. Rather than representing a discrepancy, these thresholds reflect two complementary analytical strategies commonly adopted in clinical research. Importantly, we also demonstrated that the hs-CRP/SA ratio as a continuous variable showed superior predictive performance (AUC 0.813), reinforcing the robustness of our findings independently of dichotomization. While we agree that future studies should aim to define standardized clinical thresholds, the dual approach adopted in our study provides a comprehensive evaluation of the biomarker’s prognostic value.

Role of NT-proBNP and Incremental Prognostic Value: We fully agree that NT-proBNP represents a cornerstone biomarker in heart failure, with well-established diagnostic and prognostic utility [8]. In our study, NT-proBNP was measured but not included in the multivariate model to avoid overfitting, in accordance with methodological principles related to the ratio between events and covariates. It is worth noting, however, that the primary aim of our study was not to challenge or replace established biomarkers, but rather to explore the prognostic relevance of a novel index reflecting inflammatory and nutritional status. These dimensions are only partially captured by natriuretic peptides, which primarily reflect hemodynamic stress. In this regard, the hs-CRP/SA ratio should be viewed as complementary rather than alternative to NT-proBNP. Importantly, in our cohort, NT-proBNP levels did not differ significantly between the two groups stratified by hs-CRP/SA ratio (2169.8 ± 851.3 vs. 2316.6 ± 697.3 pg/mL; *p* = 0.076), suggesting that the hs-CRP/SA ratio captures prognostic information that is not redundant with—and likely independent of—natriuretic peptide levels. This observation further supports the complementary rather than overlapping nature of these two biomarkers in risk stratification. While we agree that formal analyses assessing incremental prognostic value (e.g., NRI, IDI) would be informative, these go beyond the scope of the present study and represent a logical next step for future research, as already highlighted in our discussion.

Comparison with Individual Components: We acknowledge that competing-risk models may provide more accurate estimates in the presence of non-cardiovascular mortality. However, standard Cox regression remains a widely accepted and commonly used approach in cardiovascular outcome studies, including those involving heart failure populations. The relatively modest rate of non-cardiovascular mortality observed in our cohort suggests that the potential impact on our findings is limited. Nevertheless, we agree that competing-risk analyses may further refine risk estimation and could be considered in future studies.

External Validation and Generalizability: We recognize that the single-center design and the lack of external validation represent limitations. However, it should be noted that our cohort included a relatively large and well-characterized population of 500 patients with chronic heart failure and a long median follow-up of 5.2 years, providing robust internal validity. As with many exploratory studies, external validation is a subsequent step rather than a prerequisite for initial hypothesis generation.

Conclusions: In conclusion, we appreciate the reviewer’s comments, which have allowed us to further clarify the methodological framework and clinical relevance of our study. We believe that the hs-CRP/SA ratio represents a simple, accessible, and biologically grounded biomarker that complements established tools for risk stratification in heart failure. Indeed, serial measurements of hs-CRP/SA can improve risk stratification. Furthermore, the hs-CRP/SA ratio provides complementary prognostic information independent of natriuretic peptides. While further validation and comparative analyses are warranted, our findings provide a solid basis for future investigations and support the potential clinical utility of this integrated biomarker.

## Data Availability

No new data were created or analyzed in this study. Data sharing is not applicable to this article.
